# Percutaneous intravenous catheter forceps biopsy in right atrial mass: two case reports and literature review

**DOI:** 10.1186/s12872-022-02507-x

**Published:** 2022-02-20

**Authors:** Lei Chang, Chenyi Gong, Haitao Lu, Yihai Liu, Lina Kang, Jianzhou Chen, Lian Wang, Biao Xu

**Affiliations:** 1 Department of Cardiology, Nanjing Drum Tower Hospital, Clinical College of Nanjing Medical University, Nanjing, 210008 Jiangsu China; 2 Department of Cardiology, Nanjing Drum Tower Hospital, Nanjing University Medical School, Nanjing, 210008 Jiangsu China; 3 Department of Cardiology, Anhui Sixian Peoples Hospital, Suzhou, 234300 Anhui China

**Keywords:** Cardiac sarcoma, Intimal sarcoma, Cardiac lymphoma, Intravenous biopsy, Case report

## Abstract

**Background:**

Primary malignant tumors of the heart are rare. Although preoperative histological diagnosis is difficult, it has paramount value in therapeutic strategy development and prognostic estimation. Herein, we reported 2 cases of intracardiac tumors.

**Cases presentation:**

Both patients presented to the hospital with heart-related symptoms. Echocardiography showed massive masses in the atrium and positron emission tomography–computed tomography (PET/CT) revealed hypermetabolism and invasiveness. One patient cannot take surgery due to extensive metastasis and poor condition. The other patient was primarily diagnosed with lymphoma, and surgery was not recommended. They successfully underwent intravenous atrial biopsy, and histological samples confirmed intimal sarcoma and diffuse large B cell lymphoma. Based on immunohistochemical and molecular assessments, targeted chemotherapy was administered, resulting in clinical and imaging remission at discharge.

**Conclusions:**

Percutaneous intravenous catheter biopsy as a safe invasive test provides an accurate pathological diagnosis after imaging evaluation, and offers a therapeutic direction. Nonmalignant masses and some chemo-radiosensitive malignant tumors in the atrium could have good prognosis after targeted therapy.

## Background

Primary cardiac tumors (PCTs) are extremely rare, with an autopsy incidence ranging from 0.001 to 0.030% [1]. A 14-year population-based study revealed a prevalence for PCT of 1.38/100,000 [2]. A meta-analysis showed that the pooled prevalence of malignancies among patients diagnosed with PCT is 9.9% [3]. Primary cardiac malignancies (PCMs) still have poor prognosis, and attempts to overcome diagnostic and therapeutic difficulties are needed. The majority of PCMs are sarcomas, including angiosarcoma, rhabdomyosarcoma, fibrosarcoma, and Kaposi sarcoma. Mesotheliomas and primary cardiac lymphomas are the next most common primary cardiac malignancies [4]. Intimal sarcoma, a mesenchymal tumor, is the least reported primary cardiac tumor that originates from the tunica intima of large blood vessels, and rarely involves the heart [5]. PCT has multiple clinical presentations, ranging from asymptomatic detection in imaging tests to palpitation, shortness of breath, emaciation and even aborted sudden cardiac death [6]. Preoperative histological diagnosis is difficult but has paramount value in therapeutic strategy development and prognostic estimation. Herein, we successfully performed percutaneous atrial mass biopsy (PAMB), and established histological diagnosis instead of surgery. Safety, procedure and benefit for patients are key points for the extensive use of this invasive diagnostic technique.

## Cases presentation

**Fig. 1:**
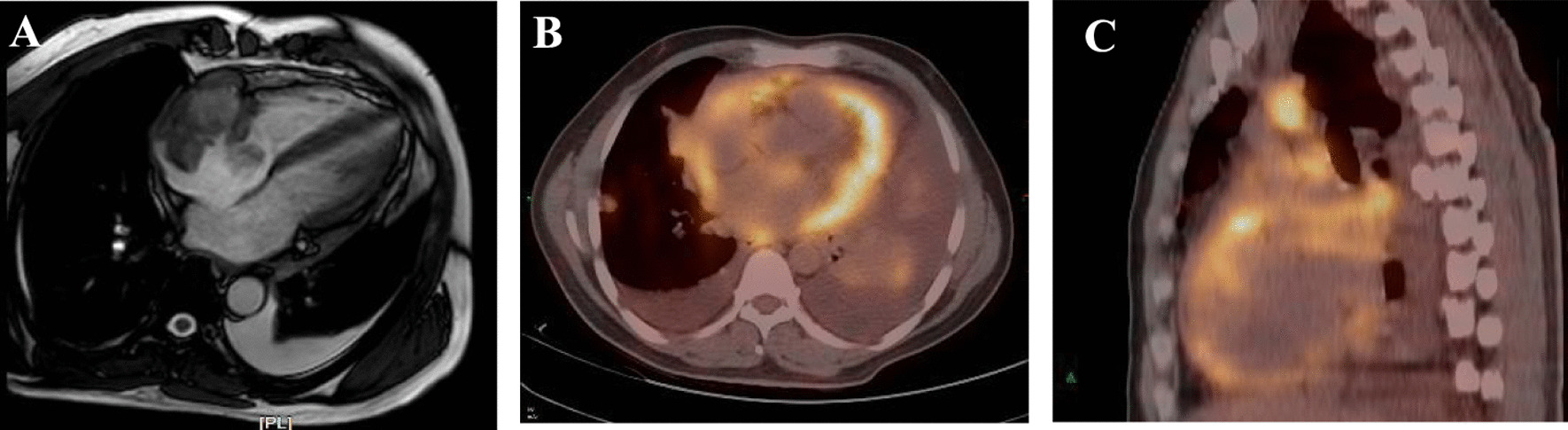
CMR shows the right atrium soft tissue mass closed to the tricuspid orifice (**a**). PET/CT scan suggests intensive tracer uptake of the right atrial mass, pericardium, lung and mediastinal lymph nodes (**b**, **c**)

**Fig. 2:**
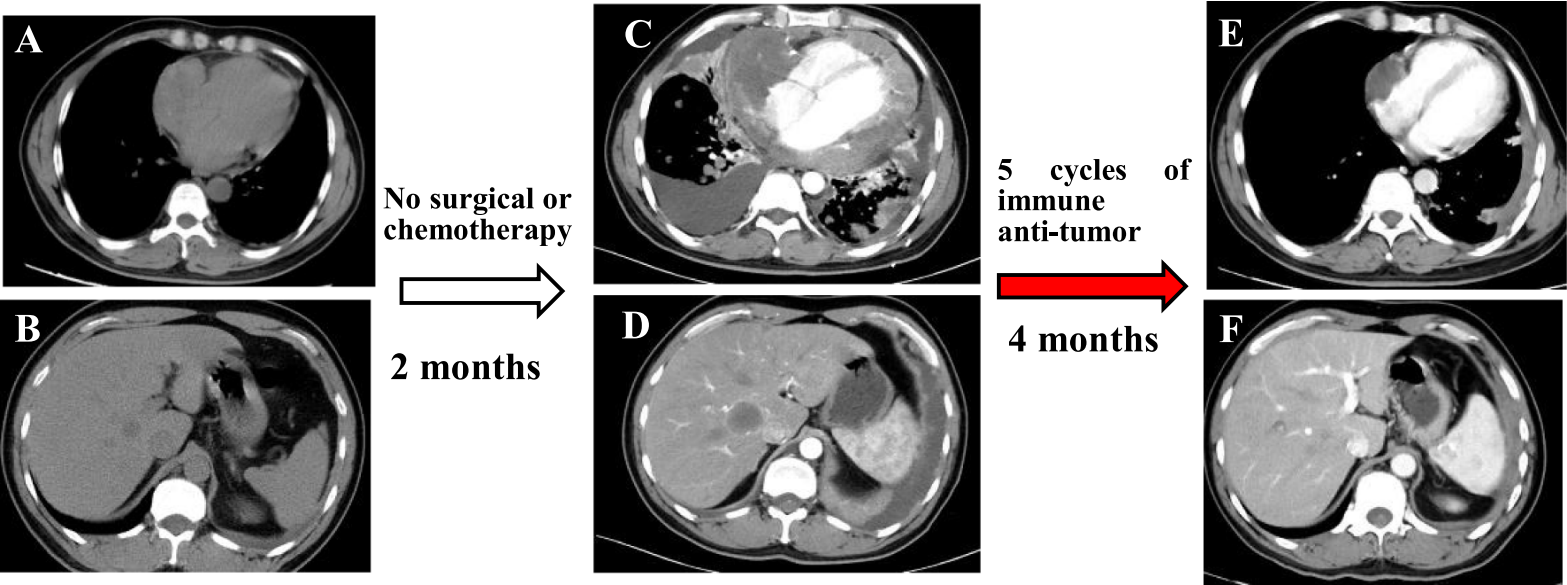
CT imaging displayed right atrium mass (**a**) and suspected metastases in liver (**b**). Scan enhancement CT scan show abnormal signals in the right atrium, pericardium (**c**), and liver (**d**) in 2 months. After 5 cycles of comprehensive immune anti-tumor therapy, significant reduction is detected in the right atrial intimal sarcoma (**e**) and liver mass (**f**) dimension

Case 1: A 48-year-old male was presented with palpitation and chest distress after activity for 3 months. Echocardiography revealed a large mass (maximum size: 58 mm × 40 mm) attached to the lateral and posterior atrial walls, invading the inferior vena cava. Cardiac magnetic resonance (CMR) imaging showed right atrial soft tissue mass close to the tricuspid orifice with the valve leaflet (Fig. 1a). Positron emission tomography-computed tomography (PET/CT) suggested enhanced fluorodeoxyglucose (FDG) metabolism signals in the right atrium, pericardium, mediastinum lymph nodes, and left lung nodules (Fig. 1b, c). Surgical resection was not recommended because of extensive metastasis. Totally 2 months later, the patient experienced severe chest tightness, wheezing, sitting breathing, lower limb edema, and bilateral bloody pleural effusion. Radiographic evaluations showed a right atrial mass and multiple metastatic lesions that had progressed (Fig. 2a–d).

**Fig. 3:**
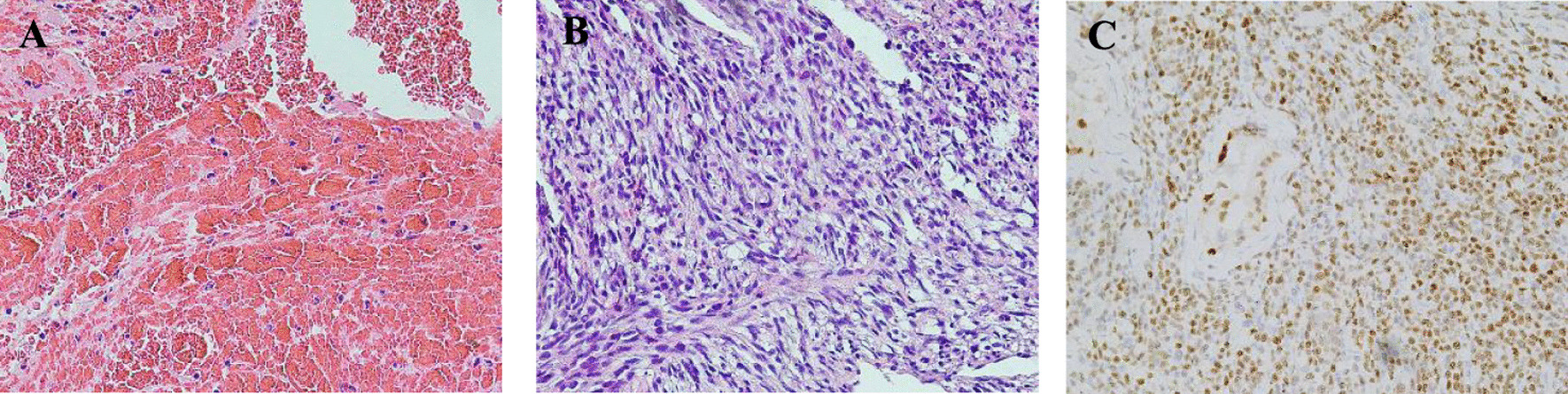
The pathology of the first biopsy via femoral vein shows a large amount of cellulose and blood cells, diagnosed as thrombus (**a** hematoxylin and eosin, 40 × 10). The pathology of the secondary biopsy through the right internal jugular vein reveals a spindle cell tumor, some areas are rich in spindle cells, with moderate atypia and focal necrosis, and the histology is consistent with intimal sarcoma (**b** hematoxylin and eosin, 40 × 10). Immunohistochemistry-positive neoplastic cells for PRAME (**c** 40 × 10)

To confirm the nature of the mass, PAMB was performed through the femoral vein, wherein three tissues were removed. The pathological analysis revealed thrombus which was consistent with imaging features. We speculated that blood flow changes caused blood turbulence. This could increase the risk of thrombus adhesion on the mass surface and influence biopsy results. After 3 weeks, the patient underwent second PAMB through the internal jugular vein. With the bedside echocardiography auxiliary positioning, three tumor tissues were removed from the atrial mass. The pathological report concluded intraepithelial metaplasia; the abnormal cell pattern showed high-grade spindle cell neoplasm with moderate atypia and focal necrosis, consistent with intimal sarcoma histologically (Fig. 3c, d). The patient was finally diagnosed with primary cardiovascular intimal sarcoma (stage IV).

Subsequently, he underwent exploratory tumor immunotherapy. The primary drug was the immune checkpoint inhibitor PD-1 antibody, which could activate endogenous anti-tumor response and has confirmed efficacy in some soft-tissue sarcomas [7]. After five cycles, the patient’s symptoms were relieved, and cardiac function was improved significantly. Re-examination by chest CT revealed a shrunk tumor in the right atrium, and the number of metastases was decreased substantially (Fig. 2e, f). At the time of drafting this manuscript, the condition of patient was stable and receiving the thirteenth cycle of immunotherapy. His health condition and life quality were improved significantly.

**Fig. 4:**
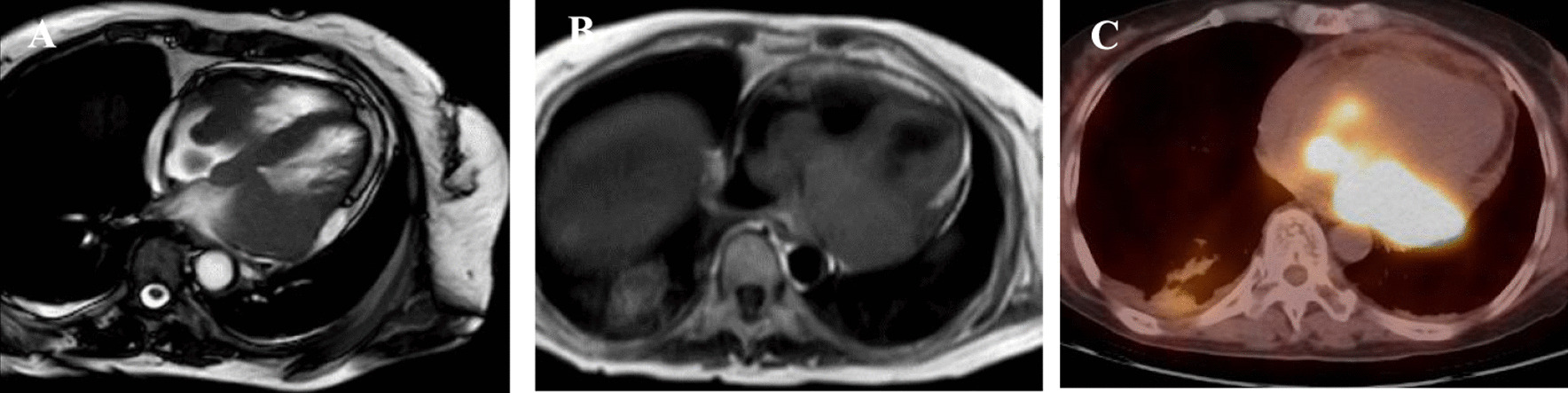
CMR confirmed left atrial mass invaded right atrium (**a**, **b**) and PET-CT scan revealed high FDG uptake in atrum and mediastinal lymph nodes (**c**)

Case 2: A 77-year-old woman presented to our hospital with a half-month history of chest tightness and dyspnea. Sinus tachycardia and pulmonary moist rales were found on physical examination. Transthoracic echocardiography revealed an intracardiac mass (maximum size: 49 mm × 38 mm) attached to the left atrium that invaded the right atrial walls and tricuspid annulus. The atrial mass protruding into the pericardial cavity caused moderate pericardial effusion and early signs of tamponade. CMR confirmed the presence of an invasive intracardiac mass (Fig. 4a, b) and PET-CT scan revealed high FDG uptake in the neoplasm and mediastinum lymph nodes (Fig. 4c). The patient underwent pericardium puncture drainage, and abnormal lymphocytes were found by pericardial effusion smear examination. Abnormally increased percent of heteromorphic monoclonal B lymphocytes was detected by flow cytometry. Given the right atrium invasion of the mass, and our successful experience of PAMB in the right atrium, we performed percutaneous jugular puncture and catheterization, and subsequent atrium angiography revealed a filling defect in the right atrium. Then, biopsy catheter was positioned at the neoplasm, and TTE provided multiple heart sections that confirmed the relative positions of biopsy forceps, atrial walls, and the mass. We successfully obtained 2 tissue pieces to avoid thoracotomy, without complication.

**Fig. 5:**
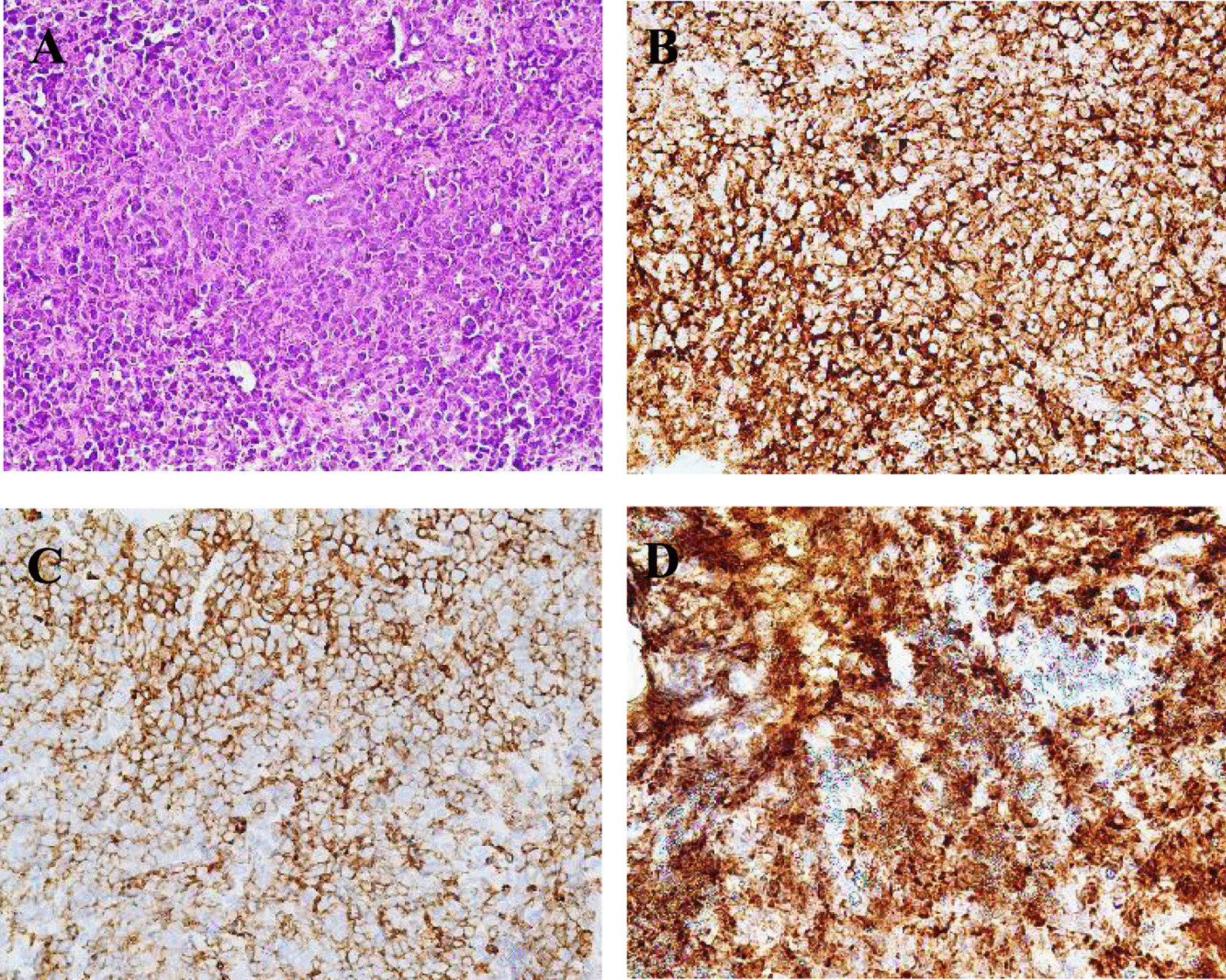
The histopathological section shows diffuse growth of large lymphocytes with a small amount of apoptotic necrosis (**a** hematoxylin and eosin, 40 × 10). Immunohistochemistry-positive neoplastic lymphoid cells for CD20 (**b** 40 × 10), CD5 (**c** × 400) and Bcl2 (**d** 40 × 10)

Immunohistochemical analysis of the specimen showed that the obtained tumor cells were positive for CD20, MUM1, CD5, and Bcl-2; negative for CD3, CD99, CD10 and CD30; and equivocalfor Bcl-6. Ki67, Bcl-2, and c-MYC labeling indexes were all 90% (Fig. 5a–d). However, bone marrow biopsy showed no infiltration of abnormal lymphocytes. Then, the patient was diagnosed with cardiac diffuse large B cell lymphoma, of non-germinal center B-cell-like type. After 3 cycles of chemotherapy with R-miniCHOP (rituximab, cyclophosphamide, adriamycin, vincristine, and prednisone), the clinical symptoms were improved remarkably. Repeated echocardiography demonstrated disappearance of the intracardiac mass. PET/CT showed no area of FDG hypermetabolism in the heart or intrathoracic lymph nodes. The patient would accept the full course of treatment with continuous follow-up.

## Discussion and conclusions

A recent multicenter analysis showed that PCM mainly affected adults (mean age of 53 years), and had a dismal long-term survival rate despite various modes of treatment [8]. The overall 1-year and 5-year survival rates were 45.3% and 11.5% in the latter study. Patients who underwent surgery had significantly better survival compared with those administered the non-surgical treatment. Moeri-Schimmel et al. indicated that most PCM patients who received postoperative radiotherapy had longer survival time than those who undergo only surgery [9]. Multi-institutional data from the National Cancer Database also confirmed that stage III PCM patients who undergo surgery and receive perioperative chemotherapy have better survival compared with those who did not receive this treatment [8]. Although surgical resection remains the first treatment choice for cardiac sarcoma [1], radiotherapy and chemotherapy are increasingly important. Thus, individualized radiotherapy and chemotherapy should be recommended in inoperable patients. A prospective study of right-sided tumors showed that neoadjuvant chemotherapy reduces the tumor burden, improving resectability and survival in complex cardiac tumors [10].

Although intracardiac mass is considered as a dangerous signal of PCM, it should be noticed that a neoplasm located in the atrium usually causes less systolic dysfunction and ventricular arrhythmia. Moreover, benign lesions such as thrombus and myxoma, accounting for a large proportion of atrial masses, but seldom appear in ventricles. Therefore, the criticality of atrial mass is different from that of ventricular mass. For atrial mass, more effort is needed to enable precise diagnosis, especially histological diagnosis. Echocardiography, CT, CMR, and PET/CT imaging could help in the preliminary differentiation between malignant and benign tumors based on tumor shape, aggressiveness, and radioactive material intake [11, 12]. However, several intra-atrial PCMs firstly misdiagnosed as benign tumors or incompletely resected may relapse in a short time [13]. Generally, histological diagnosis before tumor therapy could avoid this situation and help differentiate primary sarcomas from other malignancies (such as lymphomas that do not require surgery). After that, the patients could be administered the best-personalized multimodality treatment [14]. In patients with potential resectable cardiac sarcomas but limited by poor cardiac and pulmonary functions [15], pathological results could facilitate neoadjuvant chemotherapy to improve the basic condition of patients and create opportunities or strengthen the effect of surgical resection.

The endomyocardial biopsy technology has been widely used in cardiomyopathy, and has been extended to ventricle mass biopsy. By contrast, atrial biopsy has not been promoted for some reason. Atrial biopsy means thinner cardiac muscle walls, closer intervention to the relatively fragile vein, and higher risk of thrombus detachment. In 1989, Gosalakkal and Sugrue [16] firstly reported atrium biopsy and removed the tumor tissue from the cardiac chamber. To further evaluate the safety and efficacy of percutaneous atrial mass biopsy, we analyzed 51 cases in 47 articles and our 2 PAMB cases (Table 1). Most patients had undergone simple right atrial lump biopsy, while 3 cases underwent transseptal left atrial biopsy. Of all cases, only 1 patient reported potential complications post-biopsy [17]. The latter patient developed severe hypotensive shock after the procedure. After fluid resuscitation and treatment with vasoactive agents, the shock symptoms were stabilized. Since no obvious signs of perforation and rupture of the atrium or inferior vena cava were noted, mechanical damage to the heart caused by biopsy was not considered.

**Table1:** Atrial mass cases diagnosed by intravenous biopsy

Age	Sex	Biopsy location	Transvenous pathway	Guidance Method	Presenting reasons	Pathological diagnosis	Treatment	Outcome	PMID
14	Male	RA	Femoral vein	DSA + TEE	Chest pain, cough, and hemoptysis	/	/	/	2,334,836 [18]
46	Female	RA	Jugular vein	DSA	Symptoms of Upper respiratory infection	Metastases of melanoma	/	/	2,765,329 [16]
83	Female	RA	Femoral vein	DSA	Dyspnea and hypodynamia	Papillary fibroelastoma	Symptomatic treatment	/	2,816,690 [19]
69	Female	RA	Femoral vein	DSA + TEE	Dyspnea, edema, and syncope	Thrombus	Surgical resection	Remission	7,499,910 [20]
52	Female	RA	Femoral vein	DSA + TEE	History of cirrhosis	Metastases of liver cancer	/	Deceased	8,131,572 [21]
69	Male	RA	Femoral vein	DSA + TEE	Dyspnea, cough, and hemoptysis	Metastases of melanoma	/	/	8,154,436 [22]
69	Female	RA	Jugular vein	DSA + TEE	Dyspnea, dehydration, and ventricular tachycardia	Thrombus	Symptomatic treatment	/	8,365,328 [23]
62	Male	RA	Jugular vein	DSA + TEE	Edema and hepatic encephalopathy	Metastatic adenocarcinoma	/	Deceased	8,498,328 [24]
73	Female	RA	Jugular vein	DSA + TEE	Edema, cough, fever, and dyspnea	Lymphoma	Chemotherapy	Remission	8,579,042 [25]
69	Male	RA	Femoral vein	DSA + ICE	Flu symptoms	Metastasis of lung cancer	/	/	8,611,294 [26]
73	Male	RA	Jugular vein	DSA + TEE	Asymptomatic	Metastases of melanoma	Chemotherapy	/	8,945,488 [27]
35	Male	RA	Femoral vein	DSA + TEE	Dyspnea and cough	Angiosarcoma	Chemotherapy	Remission	8,974,823 [28]
/	/	RA	Jugular vein	DSA + TEE	/	Angiosarcoma	/	/	9,070,559 [29]
62	Male	RA	Jugular vein	DSA + TEE	Dyspnea, chest pain and	Undifferentiated sarcoma	Chemotherapy	Deceased	9,339,428 [30]
66	/	RA + AS	Femoral vein	DSA + TEE	Myocardial infarction	Lymphoma	Chemotherapy	/	9,454,452 [31]
64	Female	RA	/	DSA + TEE	Dizziness	Lymphoma	/	/	9,487,479 [32]
47	Male	RA	Jugular vein	DSA + TTE	Dyspnea, edema and pleural effusion	Lymphoma	Chemotherapy	/	9,829,904 [33]
50	Male	RA	/	DSA + TEE	Orthopnea, fever, and pleural effusion	Thrombus	Surgical resection	Deceased	9,932,633 [34]
39	Female	/	/	/	/	Myxoma	/	/	10,231,677 [35]
/	/	RA	/	/	/	/	/	/	10,763,354 [36]
77	Female	RA	/	DSA + TEE	Dyspnea, edema and hypodynamia	Lymphoma	Chemoradiotherapy	Remission	10,790,358 [37]
75	Male	RA	Femoral vein	DSA + TEE	Hemoptysis	Lymphoma	Chemotherapy	Remission	10,842,397 [38]
79	Female	RA	Femoral vein	DSA + ICE	Dyspnea and edema	Neuroblastoma	/	/	10,952,166 [39]
/	/	LA	/	DSA + TEE	/	Atrial sarcoma	/	/	11,223,492 [40]
52	Male	RA	Jugular vein	DSA + TEE	Epigastric ache	Angiosarcoma	Chemotherapy	Remission	12,019,433 [41]
62	Male	RA	Femoral vein	DSA + TEE	Dyspnea and dizziness	Lymphoma	Chemotherapy	Remission	12,848,708 [42]
64	Male	RA	Jugular vein	DSA + TEE	Heart failure	Lymphoma	Chemotherapy	Remission	14,622,547 [43]
52	Male	RA	Jugular vein	DSA + TEE	Dyspnea, chest pain, and edema	Metastases of liver cancer	Symptomatic treatment	Deceased	15,546,373 [44]
63	Female	RA	Jugular vein	DSA + ICE	Dyspnea and centrum fracture	Lipomyoma	Expectant treatment	/	16,880,106 [45]
61	Male	RA	Subclavian vein for biopsy, femoral vein for ICE	DSA + ICE	Ventricular tachycardia	Lymphoma	/	/	17,015,040 [46]
38	Male	RA	/	DSA + TTE	Dyspnea	Lymphoma	Chemotherapy	Remission	17,383,751 [47]
52	Male	RA	Femoral vein	DSA + ICE	Cardiac tamponade	Granulocytic sarcoma	Chemoradiotherapy and hematopoietic stem cell transplantation	Remission	18,498,027 [48]
56	Female	/	/	/	Repeated pericardial effusion	/	/	/	18,805,775 [49]
67	Female	RA	Jugular vein for biopsy, femoral vein for ICE	DSA + ICE	The discomfort of the precordial area	Paragangliomas	Surgical resection	/	18,818,096 [17]
47	Female	RA	Jugular vein for biopsy, femoral vein for ICE	DSA + ICE	Hypodynamia and atrial fibrillation	Paragangliomas	Surgical resection	/	18,818,096 [17]
30	Male	RA/IVC	Jugular vein for biopsy, femoral vein for ICE	DSA + ICE	Polyserositis and edema after heart transplant	Bacterial emboli	Surgical resection	Remission	18,818,096 [17]
70	Male	RA/IVC	/	/	Ascites after heart transplantation	Cardiac amyloidosis	Symptomatic treatment	Deceased	18,818,096 [17]
63	Female	RA	Jugular vein for biopsy, femoral vein for ICE	DSA + ICE	Chest discomfort and edema	Lymphoma	Chemotherapy	Remission	19,057,087 [50]
59	Male	RA	/	DSA + TEE	Dyspnea, cough, chest discomfort, fever and hypodynamia	Lymphoma	Chemotherapy	Remission	19,142,595 [51]
64	Female	RA	/	DSA + TTE	Dizziness and history of endometrial carcinoma	Metastases of endometrial adenocarcinoma	/		20,027,104 [52]
22	Female	RA	/	DSA + ICE	Dyspnea and syncope	Angiosarcoma	Chemotherapy	/	20,585,357 [53]
57	Female	RA	/	DSA + TEE	Dyspnea and back pain	Intimal sarcoma	Chemotherapy	Remission	20,966,612 [54]
44	Male	RA	Jugular vein for biopsy, femoral vein for ICE	DSA + ICE	Dyspnea	Lymphoma	Chemotherapy	Remission	22,576,384 [55]
31	Male	LA	/	DSA + TEE	Dyspnea and hypodynamia	Poorly differentiated sarcoma	Chemotherapy	Deceased	23,109,774 [56]
46	Male	RA	Femoral vein	DSA + ICE	History of myxoma	Myxoma	Expectant treatment	Remission	25,240,574 [57]
53	Male	RA	Right femoral vein for biopsy, left femoral vein for ICE	DSA + ICE	Dyspnea and weight loss	Metastases of lung cancer	Chemotherapy	Deceased	25,810,740 [58]
51	Female	RA	/	DSA + ICE	Dyspnea and edema	Angiosarcoma	Chemotherapy	Deceased	25,810,740 [58]
47	Female	RA	Femoral vein	DSA + ICE	Neoadjuvant chemotherapy for breast cancer	Thrombus	Surgical resection	Remission	27,068,834 [59]
59	Male	LA	/	DSA + TEE	Fever, weight loss, and history of HIV infection	Lymphoma	Chemotherapy	Remission	31,020,117 [60]
61	Male	RA	/	DSA + ICE	Jaundice, hypodynamia and diagnosed as IgG4/related disease	IgG4/related disease	Hormonotherapy	Remission	31,118,383 [61]
47	Female	RA	Jugular vein for biopsy, femoral vein for ICE	DSA + ICE	Cough and pulmonary nodules	Angiosarcoma	Chemotherapy	Remission	32,874,873 [62]
48	Male	RA	Femoral vein and jugular vein	DSA + TTE	Palpitation and chest discomfort	Intimal sarcoma	Chemotherapy	Remission	Present case
77	Female	RA	Jugular vein	DSA + TTE	Dyspnea and chest discomfort	Lymphoma	Chemotherapy	Remission	Present case

Among 37 cases with reported malignant tumors, lymphomas accounted for the largest proportion (15/37), followed by soft tissue sarcomas (13/37), and 9/37 reported metastatic tumors. Table 2 shows their prognoses differed from mass location and pathological results. In all patients, atrial lymphoma patients had the best prognosis, including 11 patients who presented clinical remission or even cured after receiving corresponding chemotherapy; the prognoses of another 4 lymphoma patients were not reported. Most atrial soft-tissue sarcoma patients cannot tolerate surgery and radiotherapy due to heart-related symptoms; 6 patients achieved clinical remission and 3 had tumor exacerbation in short-term visits. Of another 4 patients diagnosed with atrial thrombosis, 2 had satisfactory prognosis, and 1 diagnosed with angiosarcoma by subsequent surgical specimens died of tumor recurrence and metastasis [34]. In addition, a patient pathologically diagnosed with an IgG4-related disease received hormone therapy and showed good prognosis [61].

**Table 2:** Prognosis of patients differs with respect to the mass locations and the pathological results

Neoplasm location and pathology	Overall response	Progressive disease	Not mentioned	Total
All cases	22	9	22	53
First attack symptom				
Related to the mass	17	8	13	38
Unrelated to the mass	5	1	4	10
No discomfort or not mentioned	0	0	5	5
Biopsy location				
Right atrium or atrial septum	21	8	21	50
Involved left atrium	1	1	1	3
Malignant tumor				
Lymphoma	11	0	4	15
Metastatic tumor	0	4	5	9
Soft-tissue sarcoma	6	3	4	13
Benign tumor				
Lipomyoma	0	0	1	1
Myxoma	1	0	1	2
Paraganglioma	0	0	2	2
Fibroma	0	0	1	1
Others				
Thrombus	2	1	1	4
Bacterial vegetation	1	0	0	1
IgG4-related diseases	1	0	0	1
Myocardial amyloidosis	0	1	0	1
Not mentioned	0	0	3	3

The overall response means patients’ clinical symptoms were relieved and/or healed, or the tumor volume was reduced. Progressive disease means patients could not benefit from the treatment, it was worsened, or led to death

For various spatial atrial mass locations, different vessel approaches and ultrasonic guidance methods could be selected in PAMB. Biopsy has been performed via the internal jugular, femoral and subclavian veins. Transesophageal echocardiography (TEE) is highly effective in mass locating and bioptome guidance. Hence, about 50% of physicians use TEE as a supplementary tool to digital subtraction angiography (DSA). Transthoracic echocardiography (TTE) is more convenient but provides less location information compared with TEE, thereby necessitating highly experienced doctors. Intracardiac echocardiography (ICE) can provide the most precise intra-atrial imaging and structures, but requires an extra-intravenous ultrasound catheter, which limits its application; only about 33% of physicians utilize this method. Table 3 shows the proportion of different guiding methods in all transvenous atrium biopsy cases.

**Table 3:** Proportion of different guiding methods in all transvenous atrium biopsy cases

	Reported imaging and guidance method				Not mentioned
	DSA only	TTE + DSA	TEE + DSA	ICE + DSA	
Number of cases	2	6	24	17	4
Proportion	4.1%	12.2%	49.0%	34.7%	

Percutaneous intravenous catheter biopsy provided accurate pathological diagnosis after imaging evaluation, avoiding unnecessary surgical excision and reducing medical resource waste. Benign tumors, thrombus, bacterial vegetation, and lymphoma detected by PAMB could be cured. Nevertheless, when pathological biopsy results suggest thrombus but do not correspond to clinical symptoms, doctors should consider the possibility of false negatives. Multiple biopsies from different vessel approaches and varied mass locations might reduce the probability of misleading results. However, whether biopsies are beneficial to PCM patients is currently controversial. Ryo et al. [34] speculated that intravenous cardiac biopsy and subsequent chemotherapy is not beneficial to cardiac sarcoma patients. The core challenge is the effectiveness of subsequent anti-tumor therapy. Significantly, with the development of anti-tumor therapy, appropriate treatments have proved effective. PAMB as a safe, effective, and minimally invasive procedure for histological diagnosis before surgical resection would be applied increased comprehensively.

## Data Availability

The information and data of the 2 patients were acquired from the Hospital Information System are not publicly available due to the protection of individual privacy. The literature review datasets analyzed in the current study are available from the corresponding author on reasonable request.

## Abbreviations

PCT: Primary cardiac tumor
PCM: Primary cardiac malignancy
PET/CT: Positron emission tomography-computed tomography
CMR: Cardiac magnetic resonance
FDG: Fluorodeoxyglucose
PAMB: Percutaneous atrial mass biopsy
TEE: Transesophageal echocardiography
TTE: Transthoracic echocardiography
ICE: Intracardiac echocardiography
DSA: Digital subtraction angiography
